# Oz Virus Infection in 6 Animal Species, Including Macaques, Bears, and Companion Animals, Japan

**DOI:** 10.3201/eid3104.241574

**Published:** 2025-04

**Authors:** Aya Matsuu, Kango Tatemoto, Keita Ishijima, Ayano Nishino, Yusuke Inoue, Eunsil Park, Hiroo Tamatani, Junji Seto, Hideo Higashi, Yuichi Fukui, Takashi Noma, Kandai Doi, Rumiko Nakashita, Haruhiko Isawa, Shinji Kasai, Ken Maeda

**Affiliations:** National Institute of Infectious Diseases, Tokyo, Japan (A. Matsuu, K. Tatemoto, K. Ishijima, A. Nishino, Y. Inoue, E. Park, H. Isawa, S. Kasai, K. Maeda); Picchio Wildlife Research Center, Karuizawa, Japan (H. Tamatani); Yamagata Prefectural Institute of Public Health, Yamagata, Japan (J. Seto); Wildlife Workshop, Yamagata (H. Higashi); Komachi Animal Hospital, Tsukuba, Japan (Y. Fukui); Kubota Animal Clinic, Ozu, Japan (T. Noma); Forestry and Forest Products Research Institute, Tsukuba (K. Doi, R. Nakashita)

**Keywords:** Oz virus, viruses, tick-borne infections, vector-borne infections, zoonoses, wild animal, companion animal, Japan

## Abstract

Oz virus (OZV) was isolated from an *Amblyomma* tick in Japan and shown to cause lethal viral myocarditis in humans. However, the natural reservoirs and the distribution of OZV remain unknown. We describe epidemiologic studies conducted by using serum samples collected from mammals throughout Japan. The results showed that 27.5% of wild boars, 56.1% of Sika deer, 19.6% of Japanese macaques, and 51.0% of Asian black bears were positive for virus-neutralizing antibodies against OZV. Approximately 2.8% of dogs and 1.0% of cats also were seropositive. OZV RNA was not detected in any of the examined animal serum samples. Most seropositive animals were distributed in central and western Japan. OZV infects a wide range of animal species, including companion animals and nonhuman primates, and is distributed through central and western Japan, suggesting that further countermeasures are required to prevent this tickborne zoonotic infection.

Oz virus (OZV) is an enveloped virus belonging to the genus *Thogotovirus* of the family *Orthomyxoviridae*; the OZV genome is a minus-stranded RNA consisting of 6 segments. OZV was first isolated from an *Amblyomma testudinarium* tick collected in 2013 in Ehime Prefecture, Japan (*1*). Phylogenetic analysis has shown that this organism is classified as a Dhori virus (DHOV)–like member and is closely related to Bourbon virus (BRBV), which was isolated from a fatal human case in the United States (*2*). In a previous epidemiologic study, we detected OZV neutralization antibodies in the serum samples of wildlife (Japanese macaques, wild boars, and Sika deer) and showed that 2 of 24 hunters also were positive for those antibodies (*3*), indicating that OZV is a tickborne zoonotic pathogen. In June 2023, a female patient in her 70s in Ibaraki Prefecture, who had visited the hospital with reported fever and malaise and died of myocarditis, had OZV infection diagnosed virologically and pathologically (*4*), suggesting that OZV infection can be lethal to humans.

To our knowledge, only 2 reports on OZV have been published, and further research is needed to assess the risk for infection by this virus in Japan, such as the circulation among ticks and natural reservoirs and the risk for tick-to-human and tick-to-animal transmission. The primary objective of our study was to assess the distribution of OZV among wildlife and domestic animals by using animal samples collected on a nationwide scale. The second objective was to identify the natural reservoir of OZV in the field. The third objective was to develop an ELISA-based antibody detection system and to compare the sensitivity (in various animal species) to that of a virus-neutralization (VN) test, in the hope that the ELISA system will enable further seroepidemiologic studies and serologic diagnoses.

## Materials and Methods

### Animal Samples

We collected serum samples throughout Japan from 4 species of mammal wildlife, including 119 wild boars (*Sus scrofa*), 173 Sika deer (*Cervus nippon*), 306 Japanese macaques (*Macaca fuscata*), and 357 Asian black bears (*Ursus thibetanus*), and from 2 species of companion animals, including 249 dogs (*Canis lupus familiaris*) and 118 cats (*Felis silvestris catus*) (Table 1). Wildlife was captured either by hunters or by wildlife management teams, and sampling was approved by the respective local governments. Serum samples from 76 dogs and 76 cats were submitted to our laboratory for diagnosis of severe fever with thrombocytopenia syndrome (*5*) from veterinary clinics nationwide. We collected additional serum samples from 2 dogs and 21 cats at veterinary clinics in Ehime Prefecture, the geographic location where the virus was originally isolated (from 1 tick). We also included serum samples from 171 dogs in Ibaraki Prefecture, a geographic location where human cases of OZV infection have occurred. All animal experiments were approved by the Japan National Institute of Infectious Disease’s Institutional Animal Care and Use Committee (approval nos. 116169 and 117167).

**Table 1:** Seroprevalence of Oz virus infection in animals, Japan, 2007–2023*

Animal	Species	Years	No.	PRNT_80_			ELISA (IgG)			PRNT_80_ versus ELISA		
				No. positive	Positive rate, %		No. positive	Positive rate, %		Agreement, %	Sensitivity, %	Specificity, %
Wild boar	*Sus scrofa leucomystax*	2019–2022	119	33	27.5		33	27.5		97.1	97.7	90.9
Sika deer	*Cervus nippon*	2017–2022	173	97	56.1		69	39.9		81.5	96.1	70.1
Japanese macaque	*Macaca fuscata*	2016–2022	306	60	19.6		50	16.3		95.4	86.0	80.8
Asian black bear	*Ursus thibetanus*	2007–2021	357	182	51.0		155	43.4		85.9	95.4	80.8
Dog	*Canis lupus familiaris*	2016–2022	249	7	2.8		NA	NA		NA	NA	NA
Cat	*Felis silvestris catus*	2019–2023	97	1	1.0		NA	NA		NA	NA	NA

*PRNT_80_, 80% plaque reduction neutralization test; NA, not applicable.

### Virus

We used the EH8 strain of OZV, originally isolated from an *A. testudinarium* tick (*1*). After passaging in Vero cells (Japanese Collection of Research Bioresources Cell Bank no. JCRB9013), as described previously (*1*), we purified the EH8-2B1 strain by using a limiting dilution method. We confirmed the sequence of the resulting purified strain to be identical with that of the original EH8 (GenBank accession nos. LC320123–8).

### VN test

To perform an 80% plaque reduction neutralization test (PRNT_80_), we heat-inactivated serum samples at 56°C for 30 minutes, diluted them 1:5 with minimal essential medium (MEM) (Thermo Fisher Scientific, https://www.thermofisher.com) containing penicillin (100 unit/mL) and streptomycin (100 μg/mL) (Nacarai Tesque, https://www.nacalai.com), and preincubated them at 37°C for 1 hour with an equal volume of solution containing ≈100 PFU of the EH8-2B1 strain of OZV. We inoculated the resulting mixture into confluent monolayers of Vero cells in 24-well plates, then incubated them at 37°C for 1 hour. After incubation, we washed the cells twice with MEM containing penicillin and streptomycin, overlaid with 0.5 mL of MEM containing 0.8% agarose gel (Lonza, https://www.lonza.com) and 2% fetal bovine serum (Thermo Fisher Scientific), and cultured them at 37°C for an additional 4 days. We then fixed the cells with 10% buffered formalin (FUJIFILM Wako Pure Chemical Corporation, https://www.fujifilm.com) and stained them with 0.04% methylene blue (FUJIFILM Wako Pure Chemical Corporation). We considered plaque reductions of >80% (compared with controls) to be positive for VN antibodies. For PRNT_80_-positive samples of dogs and cats, we determined VN titers by using serum diluted from 1:10 to 1:160.

### ELISA

To detect OZV antibodies in animal serum samples, we performed ELISA by using OZV EH8-2B1–infected or mock-infected Vero cells. We inoculated the EH8-2B1 strain in Vero cells at a multiplicity of infection of 0.1/cell. For the mock antigen, we used the same volume of MEM. We incubated OZV-infected or mock-infected Vero cells for 4 days at 37°C in 5% CO_2_. After washing the cells with phosphate-buffered saline (PBS), we lysed the cells by incubation for 1 hour in radio-immunoprecipitation assay buffer (25 mmol/L Tris-HCl, 150 mmol/L sodium chloride, 1% sodium dodecyl sulfate, 1% sodium deoxycholate, and 1% Triton X-100). We then centrifuged the lysates at 12,000 × *g* at 4°C for 5 minutes; we collected and stored the resulting supernatants at –80°C. For the ELISA, we diluted the supernatants to a total protein concentration of 5 μg/mL by using 0.05 M carbonate-bicarbonate buffer (pH 9.6) and distributed them at 100 μL/well in MaxiSorp 96-well microplates (Thermo Fisher Scientific). We then incubated the plates at 4°C overnight to allow the lysates to coat the wells. We then blocked the coated plates by incubation at 37°C for 30 minutes with 200 µL/well of 1% Block Ace (Megmilk Snow Brand, https://www.meg-snow.com). We washed the blocked plates 3 times with PBS containing 0.05% Tween 20 (FUJIFILM Wako Pure Chemical Corporation) (PBS-T) and incubated them at 37°C for 30 minutes with 100 µL/well of serum diluted to 1:100 with PBS-T containing 0.4% Block Ace (Bio-Rad Laboratories, https://www.bio-rad.com). In our preliminary experiment, we confirmed that 1:100 dilution of serum samples induced low background and high sensitivity. After performing another 3 washes with PBS-T, we diluted the plates with 100 µL/well of 1 of the following horseradish peroxidase (HRP)–conjugated reagents: recombinant protein A/G (Thermo Fisher Scientific), dog IgG, dog IgM, cat IgG, or cat IgM (Fortis Life Sciences, https://www.fortislife.com). We then incubated the plate at 37°C for 30 min and then performed 3 washes with PBS-T. We distributed substrate reagent, KPL ABTS peroxidase substrate (SeraCare, https://www.seracare.com), to the plates at 100 µL/well and incubated the plates at room temperature for 30 minutes with shaking, at which point we quenched the reaction by distributing 1% sodium dodecyl sulfate (Sigma-Aldrich, https://www.sigmaaldrich.com) to the plates at 100 µL/well. We measured absorbance at a wavelength of 405 nm by using a plate spectrophotometer (Bio-Rad Laboratories). We subtracted optical density (OD) values in the mock-infected cell lysates from those in the OZV-infected cell lysates.

### Receiver Operating Characteristic Analysis

To determine the cutoff value of ELISA, we performed a receiver operating characteristic analysis between the ELISA OD values and the results of PRNT_80_ (positive or negative). For this analysis we used Prism software version 9.0 (GraphPad, https://www.graphpad.com).

### Real-Time Reverse Transcription PCR

We extracted viral RNA from 140 µL of serum samples by using the QIAamp Viral RNA mini kit (QIAGEN, https://www.qiagen.com). We conducted real-time reverse transcription PCR (RT-PCR) by using a LightCycler 480 (Roche Diagnostics, https://diagnostics.roche.com). We detected RNA sequences of OZV by using One-Step qRT-PCR QuantiTect PCR kits (QIAGEN) and virus-specific primers and probes as follows: forward primer, 5′-TCATCGACCACAACGCAGAA-3′; reverse primer, 5′-GGTCCCATCTTTGAGGGTGG-3′; probe, 5′-FAM-GCGTCCATTGTGATGGCAGCC-3′ BHQ (where FAM corresponds to 6-carboxyfluorescein fluorophore and BHQ corresponds to the Black Hole Quencher 1 [Biosearch Technologies, https://www.biosearchtech.com]). The RT-PCR cycling protocol was as follows: 50°C for 30 minutes, 95°C for 15 minutes, and 45 cycles of 95°C for 15 seconds and 60°C for 60 seconds.

### Statistical Analysis

We conducted statistical analyses by using Student *t*-test. We performed analyses in Prism software version 9.0 (GraphPad). We considered values of p<0.05 to be statistically significant.

## Results

**Figure 1:**
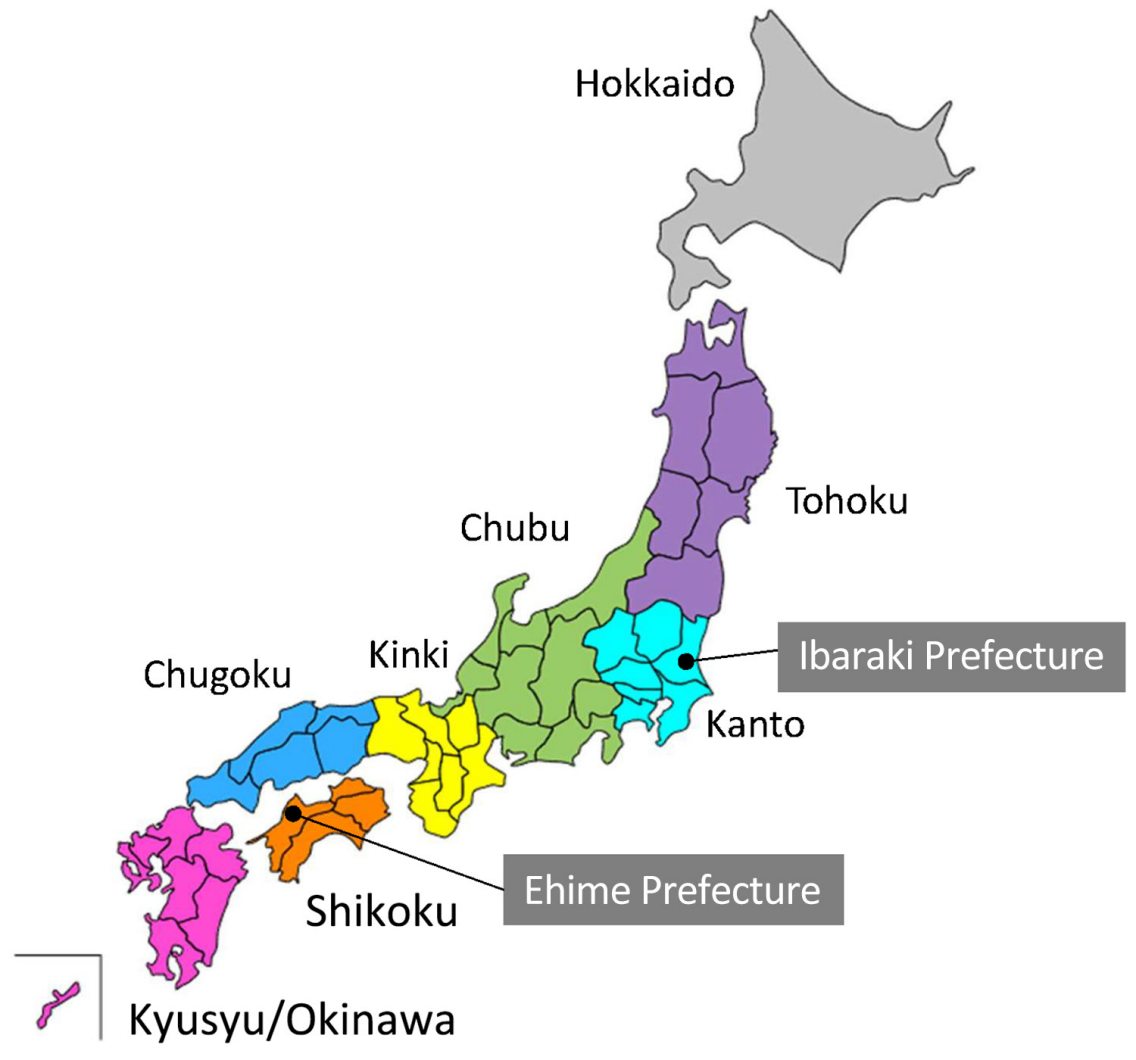
Regional divisions of Japan. Serum samples were collected from animals in all 8 regions (Hokkaido, Tohoku, Kanto, Chubu, Kinki, Chugoku, Shikoku, and Kyusyu/Okinawa) during 2007–2023. Oz virus was first isolated from an *Amblyomma testudinarium* tick obtained in the Ehime Prefecture in the Shikoku region in 2017. A fatal human case of Oz virus infection occurred in 2023 in the Ibaraki Prefecture in the Kanto region.

**Figure 2:**
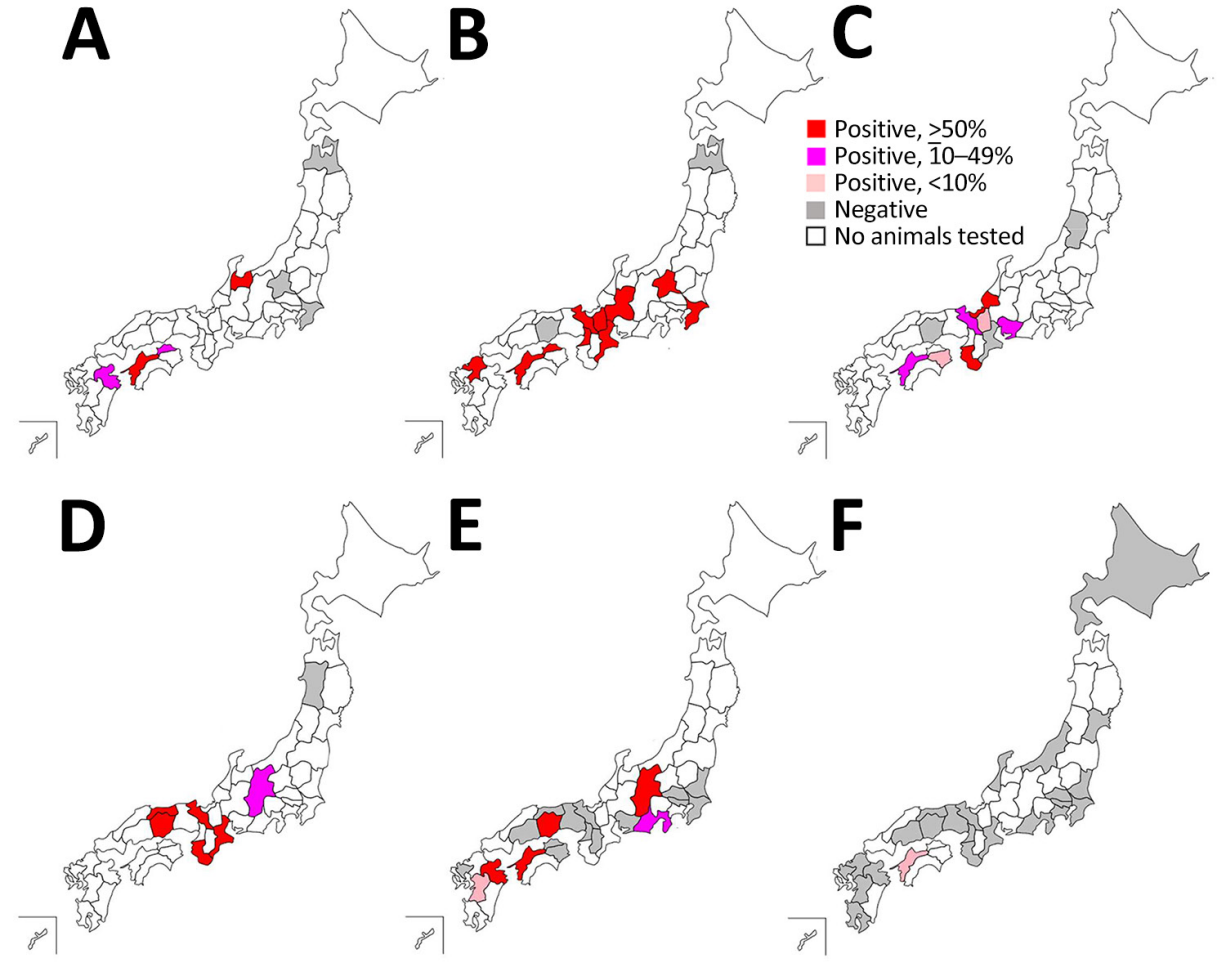
Geographic distribution of Oz virus–neutralizing antibody-positive individuals of 6 animal species, by prefecture, Japan, 2007–2023. A) Wild boar (*Sus scrofa leucomystax*). B) Sika deer (*Cervus nippon*). C) Japanese macaques (*Macaca fuscata*). D) Asian black bear (*Ursus thibetanus*). E) Dog (*Canis lupus familiaris*). F) Cat (*Felis silvestris catus*).

The results of the PRNT_80_ revealed seropositivity for OZV VN antibodies in serum samples from 33/119 (27.5%) wild boars, 97/173 (56.1%) Sika deer, 60/306 (19.6%) Japanese macaques, 182/357 (51.0%) Asian black bears, 7/249 (2.8%) dogs, and 1/97 (1.0%) cats (Table 1). We then sorted the data by geographic regions. We classified the 47 prefectures of Japan into 8 regions on the basis of geographic locations that are commonly used for administrative purposes: Hokkaido, Tohoku, Kanto, Chubu, Kansai, Chugoku, Shikoku, and Kyusyu (Figure 1). We analyzed the distribution of animals examined in this study and OZV VN antibody-positive animals (Table 2) and then visualized the data as a map (Figure 2). We found no seropositive animals in the Hokkaido and Tohoku Regions. In the Kanto Region, the Gunma Prefecture had 72.0% seropositivity and the Chiba Prefectures 54.2% among Sika deer; in this same region, all tested wild boars, dogs, and cats were negative. In the Chubu Region, we detected seropositivity in wild boars (64.3%), Sika deer (56.7%–66.7%), Japanese macaques (33.3%–61.8%), Asian black bears (21.3%–88.9%), and dogs (14.3%–50.0%). In the Kinki Region, we detected seropositivity in Sika deer (53.8%–100%), Japanese macaques (9.0%–50.0%), and Asian black bears (85.7%–100%). In the Chugoku Region, we detected seropositivity in Asian black bears (82.4%–100%) and 1 dog (50%). In prefectures in the Shikoku Region, we detected seropositivity in wild boars (20.0%–51.7%), Sika deer (60.0%–66.7%), Japanese macaques (5.9%–33.3%), dogs (100%), and 1 cat (3.2%). In the Kyusyu Region, we detected seropositivity in wild boars (19.2%), Sika deer (100%), and dogs (9.1%–50%). The most eastern region harboring seropositive animals was Chiba Prefecture; the most northern region harboring seropositive animals was Gunma Prefecture. We found no seropositive animals in parts of Japan that were farther east and north of those prefectures.

**Table 2:** Geographic distribution of virus-neutralization antibody–positive animals, Japan, 2007–2023*

Region	Prefecture	No. positive number/no. tested (positive rate, %)					
		Wild boar	Sika deer	Japanese macaque	Asian black bear	Dog	Cat
Hokkaido	Hokkaido	–	–	–	–	–	0/1 (0)
Tohoku	Aomori	0/5 (0)	0/17 (0)	–	–	–	–
	Akita	–	–	–	0/98 (0)	–	–
	Yamagata	–	–	0/56 (0)	–	–	–
	Miyagi	–	–	–	–	–	0/3 (0)
Kanto	Gunma	0/14 (0)	18/25 (72.0)	–	–	–	–
	Chiba	0/11 (0)	13/24 (54.2)	–	–	0/3 (0)	0/1 (0)
	Ibaraki	–	–	–	–	0/172 (0)	0/1 (0)
	Tokyo	–	–	–	–	0/5 (0)	0/1 (0)
	Saitama	–	–	–	–	0/2 (0)	0/5 (0)
	Kanagawa	–	–	–	–	–	0/1 (0)
Chubu	Niigata	–	–	–	–	–	0/2 (0)
	Nagano	–	–	–	16/75 (21.3)	1/2 (50.0)	–
	Shizuoka	–	–	–	–	2/14 (14.3)	0/3 (0)
	Aichi	–	–	2/6 (33.3)	–	0/2 (0)	–
	Mie	–	6/9 (66.7)	0/1 (0)	8/9 (88.9)	–	0/1 (0)
	Fukui	–	–	21/34 (61.8)	–	–	–
	Gifu	–	34/60 (56.7)	–	–	–	–
	Ishikawa	–	–	–	–	–	0/3 (0)
	Toyoma	9/14 (64.3)	–	–	–	–	–
Kinki	Shiga	–	4/4 (100)	7/78 (9.0)	–	–	–
	Kyoto	–	7/13 (53.8)	26/105 (24.8)	64/66 (97.0)	0/2 (0)	0/1 (0)
	Nara	–	–	–	18/21 (85.7)	0/1 (0)	–
	Wakayama	–	–	2/4 (50.0)	17/17 (100)	–	0/2 (0)
	Osaka	–	3/3 (100)	–	–	0/3 (0)	0/3 (0)
	Hyogo	–	–	–	–	0/18 (0)	0/11 (0)
Chugoku	Okayama	–	0/1 (0)	0/2 (0)	3/3 (100)	1/2 (50.0)	0/3 (0)
	Hiroshima	–	–	–	–	0/2 (0)	0/2 (0)
	Tottori	–	–	–	56/68 (82.4)	0/1 (0)	0/2 (0)
Shikoku	Ehime	15/29 (51.7)	2/3 (66.7)	1/3 (33.3)	–	1/1 (100)	1/31 (3.2)
	Tokushima	–	–	1/17 (5.9)	–	0/1 (0)	–
	Kagawa	4/20 (20.0)	6/10 (60.0)	–	–	0/3 (0)	–
Kyusyu	Kumamoto	–	–	–	–	1/11 (9.1)	0/1 (0)
	Oita	5/26 (19.2)	–	–	–	–	0/1 (0)
	Fukuoka	–	4/4 (100)	–	–	1/2 (50.0)	0/5 (0)
	Saga	–	–	–	–	0/2 (0)	0/2 (0)
	Kagoshima	–	–	–	–	–	0/11 (0)
Total		33/119 (27.5)	97/173 (56.1)	60/306 (19.6)	182/357 (51.0)	7/249 (2.8)	1/97 (1.0)
*–, no animals of this species were tested in this area.							

We used real-time RT-PCR to detect OZV-associated RNA in serum samples from 49 boars, 67 Sika deer, 135 Japanese macaques, 85 dogs, and 96 cats from across Japan. Of note, however, all of the assessed samples were negative.

**Figure 3:**
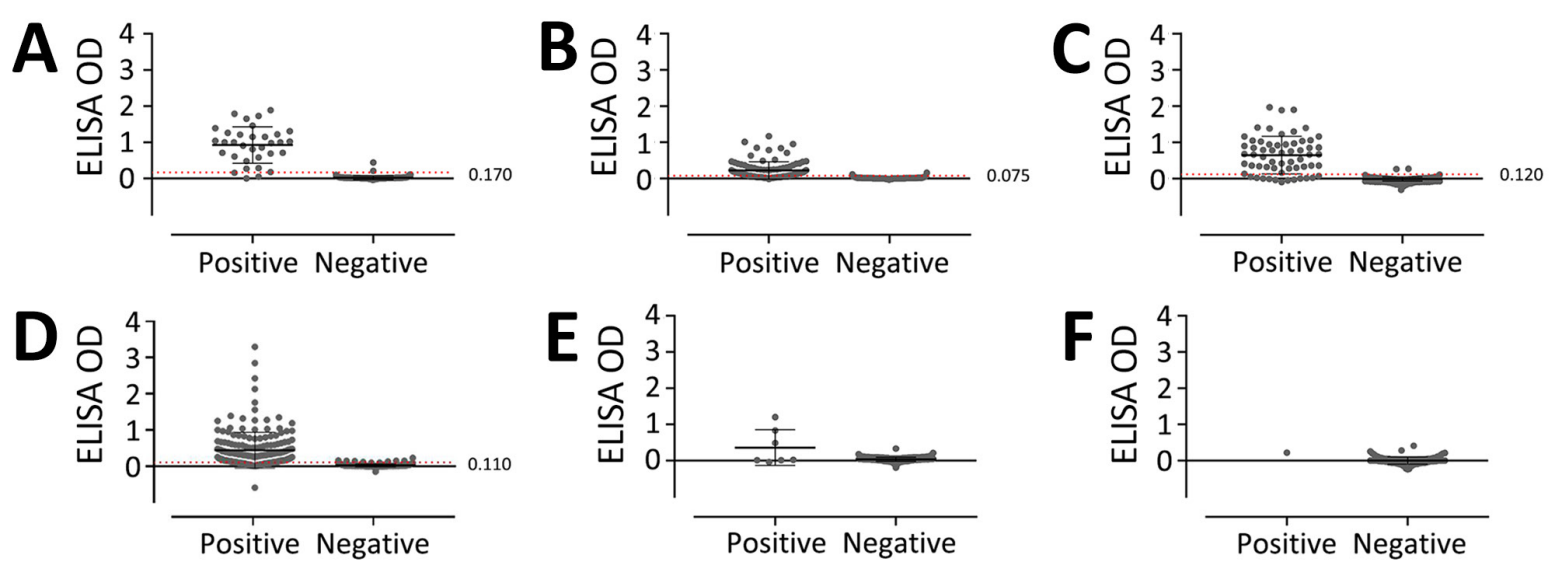
Comparison of the results of virus-neutralization tests (positive vs. negative) and ELISAs against Oz virus in serum samples from 6 tested animal species, Japan, 2007–2023. A) Wild boar (*Sus scrofa leucomystax*). B) Sika deer (*Cervus nippon*). C) Japanese macaques (*Macaca fuscata*). D) Asian black bear (*Ursus thibetanus*). E) Dog (*Canis lupus familiaris*). F) Cat (*Felis silvestris catus*). Red dashed line indicates optimal cutoff value of ELISA, which was determined by a 2-graph receiver-operating characteristic curve. The ELISA cutoff value was not determined in dogs and cats because of the low numbers of seropositive companion animals. Horizontal lines indicate means and error bars SDs. OD, optical density.

For the animal serum samples, we also conducted ELISA by using OZV EH8-2B1–infected Vero cells as a positive-control antigen, and we determined exact cutoff values for each animal species. On the basis of a receiver operating characteristic analysis, we determined the ELISA cutoff OD values to be 0.170 (97.7% specificity and 90.9% sensitivity) for wild boars, 0.075 (96.1% specificity and 70.1% sensitivity) for Sika deer, 0.120 (86.0% specificity and 80.8% sensitivity) for Japanese macaques, and 0.110 (95.4% specificity and 80.8% sensitivity) for Asian black bears (Figure 3). When we applied those cutoff values for the nationwide surveillance of OZV infection, we found that 33 wild boars (27.5%), 69 Sika deer (39.9%), 50 Japanese macaques (16.3%), and 155 Asian black bears (43.4%) had OZV antibodies by ELISA. On the basis of the cutoff values, we determined that the consistencies of judgment between the ELISA and VN tests were 97.1% in wild boars, 81.5% in Sika deer, 95.4% in Japanese macaques and 85.9% in Asian black bears (Table 1). We detected OZV IgG antibodies in dogs and 1 cat by using HRP-conjugated dog or cat IgG as secondary antibodies, but the associated OD values were low, precluding the determination of cutoff values for ELISA on samples from companion animals.

No serum samples from veterinary hospitals in the Ibaraki Prefecture tested positive for antibodies against OZV, whereas 1 dog and 1 cat from veterinary hospitals in Ehime Prefecture were seropositive (Table 3). For all dog and cat samples, we also used HRP-conjugated dog or cat IgM as secondary antibodies to detect IgM. The results showed that ELISA testing of all companion animals’ serum samples yielded OD values of <0.2 and so were judged negative. VN titers in these animals ranged from 10 to 80. All dogs and the 1 cat with VN antibodies were reported to have had access to the outdoor environment. All positive animals were mature (i.e., dogs ranged in age from 3 to 9 years and the 1 cat was 10 years of age); no sex bias was apparent. The weights of each of the VN antibody-positive dogs consistently exceeded 8.8 kg; when we compared body weight between dogs with and without VN antibodies, the VN antibody-positive dogs exhibited significantly heavier body weights (p<0.05).

**Table 3:** Summary of virus-neutralization antibody–positive dogs and cat, Japan, 2016–2023*

Animal	IgG	IgM	VN titer	Breed	Prefecture	Region	Sampling year	Age, y/sex	Body weight, kg	Rearing environment
Dog	0.83	NT	40	Setter	Kumamoto	Kyusyu/Okinawa	2020	7/M	19.2	Outdoor
	0.49	NT	>10	Unknown (hound)	Ehime	Shikoku	2020	7/UNK	Unknown	Unknown
	0.01	0.01	40	Golden retriever	Shizuoka	Chubu	2021	3/M	37.0	Outdoor
	0.01	0.04	20	Golden retriever	Okayama	Chugoku	2019	4/M	23.8	Indoor/outdoor
	0.02	0.01	10	Golden retriever	Nagano	Chubu	2021	9/M	24.5	Outdoor
	−0	0.11	40	Akita	Shizuoka	Chubu	2020	7/F	27.1	Indoor/outdoor
	1.2	NT	80	Mongrel	Fukuoka	Kyusyu/Okinawa	2020	7/F	8.8	Outdoor
Cat	0.22	0.16	20	Mongrel	Ehime	Shikoku	2019	10/UNK	Unknown	Outdoor

*NT, not tested; UNK, unknown; VN, virus neutralization.

## Discussion

Our study was a nationwide seroepidemiologic surveillance that we conducted by using serum samples from 6 animal species. We detected OZV neutralizing antibodies in individuals from all of the tested animal species; of note, the proportions of VN antibody-positive animals among companion animals were lower than those in wildlife. In addition, the geographic distribution of antibody-positive animals suggested that OZV was most widely distributed in the western part of the Kanto Region. The *A. testudinarium* tick, the species from which OZV was first isolated, also is distributed primarily in the warmer regions of Japan, a classification that includes the western part of the Kanto Region, although this tick species also has been found in Aomori and Hokkaido Regions (*6*). Therefore, the distribution of animals positive for OZV antibodies appears to correlate with the distribution of the ticks thought to serve as vectors for this virus.

The *Orthomyxoviridae* family consists of 9 species: influenza A–D viruses, *Isavirus*, *Quaranjavirus*, *Mykissvirus*, *Sardinovirus*, and *Thogotovirus* (*7*). To date, 15 genetically distinct thogotoviruses have been recognized, and infections by those viruses have been detected serologically in various mammals, suggesting that thogotoviruses have a wide host range (*8*). A range of mammals, including dogs, Eastern cottontails, horses, raccoons, and white-tailed deer in North America, have been found to possess antibodies for BRBV (positive rate 4%–86%), a virus that is phylogenetically related to OZV. Given the high prevalence of BRBV in white-tailed deer and raccoons, those animals are considered candidate reservoir species and sentinels (*9*). In our study, Sika deer and Asian black bears had the highest antibody prevalences, followed by wild boars and Japanese macaques, suggesting that Sika deer and Asian black bears might be candidate OZV reservoir species. The adult stage of the *A. testudinarium* tick has been reported to feed on large animals such as cows, wild boars, Sika deer, and bears, whereas the nymph and larvae stages appear to feed on smaller animals (*6*,*10*). In the future, seroepidemiologic surveys on small wild animals such as rodents will be necessary, as will studies to clarify the life cycle of ticks.

ELISA is widely applied for epidemiologic surveillance of viral infections among wild animals because it does not require live viruses and can detect virus-specific immunoglobulins from small amounts of serum or hemolyzed serum. In our study, we determined ELISA cutoff values for the detection of OZV antibodies in serum samples from wild boars, Sika deer, Japanese macaques, and Asian black bears. The high concordance between the PRNT_80_ and ELISA results in wild boars and Japanese macaques suggests that epidemiologic studies using ELISA are as sensitive as those using VN tests. However, concordance between the PRNT_80_ and ELISA for Asian black bears and Sika deer was lower than that for the other animal species. Those results suggested that VN test must be more suitable for seroepidemiologic surveillance of OZV infection in Sika deer and Asian black bears than ELISA.

Cross-reactivity with other closely related viruses cannot be ruled out as a cause of the high positivity rate in wildlife. In Japan, the thogotovirus-like HI-Kamigamo-25 isolate, which also has been classified as a member of the genus *Thogotovirus*, was isolated from *Hemophysalis longicornis* ticks in Kyoto, Japan (*11*). One study found that no cross-reactivity occurred between OZV and thogotovirus-like HI-Kamigamo-25 on a VN test using serum samples from experimentally infected mice and that OZV cross-reacted only with BRBV antibody by VN test (*12*). It is unknown which virus (OZV or an OZV-like virus) infects antibody-positive animals. Further study will be required to determine the viruses infecting wild animals.

In the human case, OZV infection caused severe myocarditis (*4*). Although OZV might have high virulence in humans, its infectivity and risk for infection in humans are still unknown. One previous study has shown that intracerebral inoculation of OZV is lethal for suckling mice (*1*), whereas another study showed that intraperitoneal administration of OZV to adult mice does not cause weight loss or death (*12*). In our study, we analyzed serum samples from dogs and cats with fever, leukopenia, and thrombocytopenia. In all cases, the levels of OZV IgM were not elevated, and OZV-associated RNAs could not be detected in serum samples from these companion animals. Further analysis will be needed to understand the pathogenicity of OZV in mammals, including humans.

In conclusion, we used serum samples from 6 different animal species in Japan to investigate the seroprevalence of OZV infection. We found that the OZV infects many of the wild animals that inhabit the areas from the Kanto Region in the west of Japan. We also observed infections of companion animals, although rates of seropositivity were lower than those observed in wild animals.
